# Catalytic Liquid-Phase Oxidation of Phenolic Compounds Using Ceria-Zirconia Based Catalysts

**DOI:** 10.3389/fchem.2018.00553

**Published:** 2018-11-15

**Authors:** Naoyoshi Nunotani, Abdul Rohman Supandi, Pil-Gyu Choi, Nobuhito Imanaka

**Affiliations:** Department of Applied Chemistry, Faculty of Engineering, Osaka University, Suita, Japan

**Keywords:** catalyst, liquid-phase oxidation, phenolic compounds, ceria-zirconia, catalytic wet air oxidation

## Abstract

Catalytic liquid-phase oxidation using a catalyst and oxygen gas (Catalytic wet air oxidation, CWAO) is one of the most promising technology to remove hazardous organic compounds in wastewater. Up to now, various heterogeneous catalysts have been reported for phenolic compounds decomposition. The CeO_2_-ZrO_2_ based catalysts have been recently studied, because CeO_2_-ZrO_2_ works as a promoter which supplies active oxygen species from inside the lattice to the active sites. Since it is difficult to dissolve oxygen gas into water, the use of the promoter is effective for realizing the high catalytic activity at moderate conditions. Also, CeO_2_-ZrO_2_ shows high resistance for the metal leaching during the catalytic reaction in the liquid-phase. This article reviews the studies of the catalytic liquid-phase oxidation of phenolic compounds using CeO_2_-ZrO_2_ based catalysts.

## Introduction

To date, industrial sector has been rapidly growing and consequently contributing to an increase in hazardous waste. Phenolic compounds, e.g., phenol, chlorophenol, and bisphenol-A, are well-known organic pollutants encountered in the wastewater. They have been used as raw material in many kinds of industries for the production, such as phenol resins, polycarbonate plastics, and acetylsalicylic acid. However, phenolic compounds are toxic substances for human health and lethal to aquatic life in water. Especially, bisphenol-A has identified as an endocrine-disrupting chemical (EDC) and functions as an estrogenic substance in living organisms even at very low concentration level (Geens et al., 2012). To protect the environment and our health, it is necessary to remove these pollutants from wastewater.

The conventional treatments are activated carbon treatment, coagulating sedimentation process, and biodegradation process (Chung et al., 2003; Villegas et al., 2016; Karri et al., 2017); however, these processes have problems, such as replacement of activated carbon, post-treatment of sediments, and control of temperature and pH for microorganisms. Although advanced treatment technologies have been studied using UV irradiation, strong oxidizing agents (H_2_O_2_ or O_3_), Fenton reagent (H_2_O_2_ + Fe^3+^), and ultrasound (Rosenfeldt and Linden, 2004; Torres et al., 2008; Nidheesh, 2015; Zhang et al., 2018), they require constant supply of hazardous oxidizing additives, UV irradiation, or ultrasonic irradiation.

A liquid-phase oxidation with a gaseous oxygen, wet air oxidation (WAO), is the simple process for wastewater treatment without photoirradiation, sonication, nor addition of oxidizing agent. However, the effective removal requires elevated temperatures (125–320°C) and high pressures (0.5–20 MPa) in order to increase the solubility of oxygen molecule in the solution (Zimmermann, 1958; Li et al., 1991; Mishra et al., 1995). One of the most promising processes to realize the high efficiency under the mild conditions is a catalytic liquid-phase oxidation, generally denoted as catalytic wet air oxidation (CWAO). Homogeneous catalysts based on copper salts are reported to exhibit effective catalytic oxidation for treating wastewater (Imamura et al., 1982; Kulkarni and Dixit, 1991; Lin et al., 1996); however, the dissolved catalysts must be separated due to the toxicity of copper ions. Therefore, heterogeneous catalysts have received attention, because the additional separation process is not necessary. For the heterogeneous catalysts, metal oxides and supported noble metals have been extensively studied. Among the metal oxide catalysts, such as CuO, ZnO, and MnO_2_ (Katzer et al., 1976; Pintar and Levec, 1994; Chen et al., 2001; Santos et al., 2005), CeO_2_-based catalysts are effective for the oxidation of organic pollutants. In particular, CeO_2_-MnO_2_ solid solutions exhibited the high catalytic activity for decomposing organic compounds (Imamura et al., 1985, 1987; Ma et al., 2017), while these catalysts were deactivated because of the leaching of the active phase and the formation of carbonaceous deposits, likely due to the presence of manganese (Delgado et al., 2006). Noble metals (Pt, Ru, and Pd) have generally high catalytic activity and high resistance for metal leaching compared to metal oxides (Imamura et al., 1988; Masende et al., 2005). However, they still require severe reaction conditions compared to ambient temperature and pressure. Since it is difficult to dissolve oxygen gas into water at moderate conditions, a promoter which supplies active oxygen species from its lattice toward an activator is an important component. Recently, ceria-zirconia (CeO_2_-ZrO_2_) based catalysts have been paid attention for the wastewater treatment due to their high oxygen release and storage abilities.

In this mini-review, we provided the studies on the heterogeneous catalysts based on CeO_2_-ZrO_2_ solid solutions for catalytic liquid-phase oxidation of phenolic compounds.

## CeO_2_-ZrO_2_ solid solutions

CeO_2_ is one of the most famous promoters, because of its unique non-stoichiometric characteristics resulting from the ability of easily transition between reduced and oxidized states (Ce^3+^⇄ Ce^4+^ + e^−^) (Trovarelli, 1996; Montini et al., 2016). This allows CeO_2_ to store gaseous oxygen into its crystal lattice and subsequently release active oxygen from the bulk material. Therefore, CeO_2_ can work as a promoter to facilitate the oxidation of organic compounds by supplying active oxygen to the activator. Zirconium ions (Zr^4+^) are generally introduced into the CeO_2_ lattice to increase the oxygen release and storage abilities (Fornasiero et al., 1995). In the CeO_2_-ZrO_2_ binary system, various crystal structure have been reported. Yashima et al. proposed the phase diagram of the CeO_2_-ZrO_2_ system by using X-ray powder diffraction measurement and Raman spectroscopy (Yashima et al., 1994; Varez et al., 2006; Montini et al., 2016). The phase diagram shows three different tetragonal phases (*t, t*′, and *t*^′′^), in addition to the monoclinic ZrO_2_ phase and the cubic CeO_2_ phase. The monoclinic and/or *t* phases were formed for CeO_2_ content less than ca. 20 mol%, where the monoclinic phase is thermodynamically stable for pure ZrO_2_ at room temperature. In the CeO_2_ rich area above 90 mol%, the cubic phase was detected. The *t*′ and *t*^′′^ phases were regarded as the metastable phase at intermediate compositions of 20–90 mol% CeO_2_. For the 50 mol% CeO_2_ content, other metastable structure has been identified to be pyrochlore, κ and t^*^ phases, which are cation-ordered phases (Montini et al., 2016). According to Fornasiero et al. (1995), the highest oxygen storage value is found for the Rh supported on cubic Ce_0.5_Zr_0.5_O_2_ sample. By applying the oxygen release and storage abilities, the CeO_2_-ZrO_2_ solid solution has been commercialized as promoters in automotive exhaust catalysts.

## Catalytic liquid-phase oxidation of phenol

As described in the Introduction section, phenol has received attention due to its toxicity and common pollutant in wastewater. In addition, since phenol is the simplest class among phenolic compounds, phenol is considered to be an intermediate in the catalytic oxidation of phenolic compounds. Therefore, phenol is generally selected as a model compound for the wastewater treatment using heterogeneous catalysts.

For catalytic phenol oxidation, noble metal loaded catalysts have been extensively studied due to their high catalytic activity and high resistance for the metal leaching. As for the noble metal activator, Barbier et al. (2005) investigated the Ru, Pd, or Pt supported on CeO_2_ catalysts, and confirmed that the order of phenol conversion was: Ru > Pd > Pt. For the Pd activator, the abrupt deactivation was also observed due to the deposition of carbonaceous species. Lee et al. (2010) studied the deactivation of catalyst during the phenol oxidation, and demonstrated that the deactivation was accelerated especially for Pt/Al_2_O_3_ compared to Pt/CeO_2_. They discussed that CeO_2_ might have promoted the oxidation of the carbonaceous deposits on the catalyst. According to Nousir et al. (2008), when introducing ZrO_2_ into the CeO_2_ lattice, oxygen storage capacity (OSC) of Pt/Ce_0.9_Zr_0.1_O_2_ was significantly enhanced compared to that of Pt/CeO_2_, indicating an increase in the oxygen species mobility. Although the phenol conversion of Pt/Ce_0.9_Zr_0.1_O_2_ (92%) was comparable with that of Pt/CeO_2_ (94%) after the reaction at 160°C and 2 MPa for 3 h, the selectivity in the complete oxidation product (CO_2_) for Pt/Ce_0.9_Zr_0.1_O_2_ (61%) was higher than that for Pt/CeO_2_ (50%); i.e., the formation of carbonaceous species on Pt/Ce_0.9_Zr_0.1_O_2_ was suppressed compared to the Pt/CeO_2_ case.

Based on the results mentioned above, the catalytic liquid-phase oxidation activities of phenol over the CeO_2_-ZrO_2_ based catalysts are tabulated in Table 1. Nousir et al. (2008) also compared the Pt/Ce_1−x_Zr_*x*_O_2_ (*x* = 0.9, 0.75, 0.5) catalysts, and demonstrated that the phenol conversion was: Pt/Ce_0.9_Zr_0.1_O_2_ (92%) > Pt/Ce_0.5_Zr_0.5_O_2_ (80%) > Pt/Ce_0.75_Zr_0.25_O_2_ (67%), and the CO_2_ selectivity was: Pt/Ce_0.5_Zr_0.5_O_2_ (71%) > Pt/Ce_0.9_Zr_0.1_O_2_ (61%) > Pt/Ce_0.75_Zr_0.25_O_2_ (53%). Among them, the Pt/Ce_0.5_Zr_0.5_O_2_ catalyst exhibited the highest OSC value, which caused the high CO_2_ selectivity due to an easier elimination of the surface carbonaceous deposits. In addition, they assumed that the selectivity was also affected by the large interface between Pt and the support for Pt/Ce_0.5_Zr_0.5_O_2_. Keav et al. (2014) reported that Ru supported on Zr_0.1_Ce_0.9_O_2_ showed the higher phenol conversion (ca. 97%) than the Pt supported case (ca. 92%) after the reaction at 160°C and 2 MPa for 3 h, similar to the pure ceria supported catalysts (Barbier et al., 2005). They also demonstrated that Zr_0.1_(Ce_0.75_Pr_0.25_)_0.9_O_2_ exhibited the high OSC value compared to Zr_0.1_Ce_0.9_O_2_, because two kinds of valence states (Pr^3+^ and Pr^4+^) modified the kinetics of oxygen transfer, leading to promoting the redox process. However, the phenol conversions using Pt or Ru supported on Zr_0.1_(Ce_0.75_Pr_0.25_)_0.9_O_2_ was lower than each catalyst without Pr ion (Table 1), due to the formation of carbonaceous deposits. One possible reason of the low activity was the reduction treatment using hydrogen gas (30 mL·min^−1^) at 350–800°C for 3 h before the catalytic phenol oxidation. According to Wang et al. (2008), a complete removal of phenol was realized for a Ru/ZrO_2_-CeO_2_ (Ce/Zr = 9) catalyst in the fixed-bed flow reactor by applying conditions of 4 MPa and 140°C. In a recent study, the effective phenol removal was demonstrated even at the moderate conditions by using the catalyst, which consists of Pt and Ce_0.68_Zr_0.17_Sn_0.15_O_2_ dispersed on mesoporous silica SBA-16 (Santa Barbara Amorphous No. 16) with large surface area (Supandi et al., 2017). They reported that the phenol conversion of Pt/Ce_0.68_Zr_0.17_Sn_0.15_O_2_/SBA-16 reached up to 91% after the reaction at moderate temperature of 80°C and the atmospheric pressure for 6 h. Here, only ca. 4% of phenol was adsorbed into the SBA-16 support, indicating that the catalytic reaction was proceeded. They also revealed that the conversion of Pt/Ce_0.68_Zr_0.17_Sn_0.15_O_2_/SBA-16 was also higher than that of Pt/Ce_0.8_Zr_0.2_O_2_/SBA-16 (85%). Based on the literature (Yasuda et al., 2012), the introduction of SnO_2_ into the CeO_2_-ZrO_2_ lattice enhanced the oxygen release and storage abilities, because of the synergistically redox reaction between Ce^4+/3+^ and Sn^4+/2+^.

**Table 1:** Catalytic oxidation of phenol by using CeO_2_-ZrO_2_ based catalysts.

**Catalyst**	**Removal percentage / %**	**Reaction conditions**	**References**
Pt/Ce_0.9_Zr_0.1_O_2_	92	Batch type (autoclave), 160°C, 2 MPa (O_2_)^*1^, 3 h	Nousir et al., 2008
Pt/Ce_0.75_Zr_0.25_O_2_	67		
Pt/Ce_0.5_Zr_0.5_O_2_	80		
Ru on Zr_0.1_Ce_0.9_O_2_	97	Batch type (autoclave), 160°C, 2 MPa (O_2_)^*1^, 3 h	Keav et al., 2014
Pt on Zr_0.1_Ce_0.9_O_2_	92		
Ru on Zr_0.1_(Ce_0.75_Pr_0.25_)_0.9_O_2_	95		
Pt on Zr_0.1_(Ce_0.75_Pr_0.25_)_0.9_O_2_	76		
Ru/ZrO_2_-CeO_2_ (Ce/Zr = 9)	100	Fixed-bed type, 140°C, 4 MPa (air), 80 mL·min^−1^	Wang et al., 2008
Pt/Ce_0.8_Zr_0.2_O_2_/SBA-16	85	Batch type (open-system), 80°C, 6 h	Supandi et al., 2017
Pt/Ce_0.68_Zr_0.17_Sn_0.15_O_2_/SBA-16	91		
Ce_0.68_Zr_0.32_O_2_	98.2	Batch type (autoclave), 160°C, 2 MPa (O_2_)^*2^, 7 h	Delgado et al., 2012
CuO*_*x*_*/Ce_0.65_Zr_0.35_O_2_	100	Batch type (autoclave), 150°C, 5 MPa (air)^*1^, 3 h	Kim et al., 2009
MnO*_*x*_*/Ce_0.65_Zr_0.35_O_2_	94		
NiO*_*x*_*/Ce_0.65_Zr_0.35_O_2_	69		
CoO*_*x*_*/Ce_0.65_Zr_0.35_O_2_	53		
FeO*_*x*_*/Ce_0.65_Zr_0.35_O_2_	45		
CuO/CeO_2_-ZrO_2_ (Ce/Zr = 3)	100	Batch type (autoclave), 160°C, 0.1 MPa (O_2_)^*2^, 3 h	Parvas et al., 2014b
Ni/CeO_2_-ZrO_2_ (Ce/Zr = 3)	29.5	Batch type (autoclave), 160°C, 0.1 MPa (O_2_)^*2^, 3 h	Parvas et al., 2014a

* 1 *Partial pressure under the catalytic reaction*.

* 2 *Initial partial pressure before heating the autoclave*.

Noble-metal free catalysts for phenol removal have been also studied, as listed in Table 1. Delgado et al. (2012) investigated the catalytic activity of CeO_2_-ZrO_2_ without activators. Compared to pure CeO_2_, Ce_0.68_Zr_0.32_O_2_ showed the high performance, where the phenol conversions of CeO_2_ and Ce_0.68_Zr_0.32_O_2_ were 88.4 and 98.2%, respectively, after the reaction at 160°C and 2 MPa for 7 h. The enhancement in the activity was explained by the increase in oxide ion mobility; i.e., the vacancies or structural defects might form by the incorporation of Zr^4+^ into the fluorite CeO_2_ structure. In the case of the Zr-rich sample (Ce_0.15_Zr_0.85_O_2_), the activity decreased to 89.6% due to the segregation of ZrO_2_. They also reported that a carbonaceous deposit was not observed after the reaction at 160°C, while the deposit covered the catalyst surface in the case of the reaction at 120°C, resulting the deactivation (phenol conversion of Ce_0.68_Zr_0.32_O_2_: 60.9%). In addition, they revealed the high resistance for metal leaching of CeO_2_-ZrO_2_ after the reaction. Kim et al. (2009) developed various CeO_2_-ZrO_2_ supported metal oxide activators, and demonstrated that CuO_*x*_/Ce_0.65_Zr_0.35_O_2_ showed the high catalytic activity for phenol oxidation compared to MnO_*x*_/Ce_0.65_Zr_0.35_O_2_, FeO_*x*_/Ce_0.65_Zr_0.35_O_2_, CoO_*x*_/Ce_0.65_Zr_0.35_O_2_, and NiO_*x*_/Ce_0.65_Zr_0.35_O_2_. The complete phenol conversion was confirmed for CuO_*x*_/Ce_0.65_Zr_0.35_O_2_ after the reaction at 150°C under 5 MPa for 3 h. The high activity of CuO_*x*_/Ce_0.65_Zr_0.35_O_2_ was attributed to the leached copper ions, which caused a homogeneous oxidation reaction. In the case of MnO_*x*_/Ce_0.65_Zr_0.35_O_2_, while the high conversion of phenol (94%) was obtained, phenol was mainly converted to carbonaceous deposits on the surface of the catalyst, which caused the deactivation. The high activity for the CuO supported on CeO_2_-ZrO_2_ was also reported by Parvas et al. (2014b), while Ni/CeO_2_-ZrO_2_ showed low activity (Parvas et al., 2014a).

## Catalytic liquid-phase oxidation of phenol derivatives

Up to date, effective oxidation of phenol derivatives has also been demonstrated by using CeO_2_-ZrO_2_ based catalysts, and their activities are summarized in Table 2. Li et al. (2007) studied the oxidation of *o*-chlorophenol over Ru/CeO_2_-ZrO_2_ solid solutions. The Ru/CeO_2_-ZrO_2_ catalysts have higher catalytic activities than Ru/CeO_2_ and Ru/ZrO_2_, because the oxygen supply to the active site from liquid phase was expected to be accelerated, where CeO_2_ supported catalyst might also be affected by the formation of carbonates at the catalyst surface. Among the Ru/CeO_2_-ZrO_2_ systems, Ru/Ce_0.16_Zr_0.84_O_2_ showed the highest activity, and the complete *o*-chlorophenol conversion was realized (140°C, total pressure 3 MPa, 6 h). They also reported that the introduction of a small amount of Pr or Nd into the CeO_2_-ZrO_2_ lattice enhanced the catalytic activity, because of the formation of oxygen vacancies, which increased the reducibility and facilitated the oxygen mobility. In particular, Ru/Ce_0.33_Zr_0.63_Pr_0.04_O_2_ showed the high total organic carbon removal of 95% compared to the Ru/Ce_0.16_Zr_0.84_O_2_ case (91%) (140°C, total pressure 3 MPa, 6 h), while both catalysts could completely convert *o*-chlorophenol. In the absence of Ru, Ce_0.62_Zr_0.38_O_2_ exhibited the high *o*-chlorophenol conversion of 99% under the severe conditions (140°C, total pressure 5 MPa, 24 h).

**Table 2:** Catalytic oxidation of phenol derivatives by using CeO_2_-ZrO_2_ based catalysts.

**Catalyst**	**Phenol derivatives**	**Removal percentage / %**	**Reaction conditions**	**References**
Ru/Ce_0.16_Zr_0.84_O_2_	*o*-Chlorophenol	100	Batch type (autoclave), 140°C, 3 MPa (total)^*1^, 6 h	Li et al., 2007
Ru/Ce_0.33_Zr_0.63_Pr_0.04_O_2_	*o*-Chlorophenol	100	
Ce_0.62_Zr_0.38_O_2_	*o*-Chlorophenol	99	Batch type (autoclave), 140°C, 5 MPa (total)^*1^, 0.9 MPa (O_2_)^*2^, 24 h
Ag/Ce_0.85_Zr_0.15_O_2_	Bisphenol-A	76	Batch type (autoclave), 160°C, 2 MPa (air)^*2^, 3 h	Heponiemi et al., 2015
Ce_0.67_Zr_0.18_Bi_0.15_O_2_/SBA-16	Bisphenol-A	86	Batch type (open-system), 80°C, 3 h	Choi et al., 2017
Ce_0.85_Zr_0.15_O_2_/SBA-16	Bisphenol-A	60	

* 1 *Total pressure under the catalytic reaction*.

* 2 *Partial pressure under the catalytic reaction*.

For the catalytic oxidation of bisphenol-A, most of catalysts were used in combination with strong oxidizing reagent (H_2_O_2_ etc.) or UV irradiation (Ohko et al., 2001; Mayani et al., 2014; Liu et al., 2016; Zhou et al., 2016). Heponiemi et al. (2015) reported the catalytic decomposition of bisphenol-A using Ag supported on CeO_2_-based catalysts. The bisphenol-A abatement of Ag/CeO_2_ was 51% after the reaction at 160°C for 3 h under a pressure of 2 MPa, where ca. 12% of bisphenol-A was adsorbed on the catalyst. By adding a small amount of ZrO_2_ to CeO_2_, the bisphenol-A removal percentage was enhanced; i.e., the removal percentage of Ag/Ce_0.85_Zr_0.15_O_2_ was 76% with 1% adsorption of bisphenol-A at the same condition. Choi et al. (2017) demonstrated that the CeO_2_-ZrO_2_-Bi_2_O_3_ supported on SBA-16 catalyst exhibited the catalytic activity for bisphenol-A oxidation, and 86% of bisphenol-A conversion was achieved for Ce_0.67_Zr_0.18_Bi_0.15_O_2_/SBA-16 even with the moderate reaction conditions of 80°C for 3 h under atmospheric pressure. Here, this conversion of Ce_0.67_Zr_0.18_Bi_0.15_O_2_/SBA-16 was higher than that of Ce_0.85_Zr_0.15_O_2_/SBA-16 (60%). This enhancement was explained by the high oxygen release and storage abilities of Ce_0.67_Zr_0.18_Bi_0.15_O_2_, because the introduction of Bi_2_O_3_ into the CeO_2_-ZrO_2_ lattice facilitated the oxygen supply via oxygen vacancies, formed by the replacement of Ce^4+^ and Zr^4+^ sites by the lower-valent Bi^3+^ ion (Imanaka et al., 2007). Choi et al. (2017) also reported that the Ce_0.67_Zr_0.18_Bi_0.15_O_2_/SBA-16 catalyst maintained a high conversion after three consecutive reactions without the deactivation.

## Conclusion and perspectives

In this mini-review, recent studies on catalytic liquid-phase oxidation of phenolic compounds using CeO_2_-ZrO_2_ based catalysts are summarized. By using CeO_2_-ZrO_2_ as a promoter, the catalytic activity was facilitated compared to the bare oxides, such as Al_2_O_3_ and CeO_2_. Pt/CeO_2_-ZrO_2_ and Ru/CeO_2_-ZrO_2_ catalysts exhibited high activities for phenol conversion, and the complete phenol decomposition was realized for the Ru/ZrO_2_-CeO_2_ (Ce/Zr = 9) catalyst under conditions of 140°C and 4 MPa. Even at the moderate conditions of 80°C and atmospheric pressure, the high phenol conversion of 91% was demonstrated by dispersing Pt and CeO_2_-ZrO_2_-SnO_2_ into SBA-16. Noble metal free catalysts have been also developed, and phenol was completely removed using the CuO_*x*_/CeO_2_-ZrO_2_ catalyst, in which the leached Cu^2+^ ions promoted the phenol oxidation by work as a homogeneous catalyst.

CeO_2_-ZrO_2_ was also effective for the removal of phenol derivatives. Ru/Ce_0.33_Zr_0.63_Pr_0.04_O_2_ completely converted *o*-chlorophenol, and the total organic carbon removal of 95% was realized. For the bisphenol-A removal, the conversions of Ag/CeO_2_-ZrO_2_ and CeO_2_-ZrO_2_-Bi_2_O_3_/SBA-16 were 76% (reaction conditions: 160°C, 2 MPa, 3 h) and 86% (reaction conditions: 80°C, atmospheric pressure, 3 h), respectively.

For future perspective, it is necessary to realize complete oxidation of phenolic compounds in facile operating conditions at room temperature and under atmospheric pressure, from the viewpoint of economically, safety, and sustainably. In addition, the suppression of the deactivation, caused by the carbonaceous deposit and the metal leaching, should be considered. Since one effective method to remove the carbonaceous deposit is the complete oxidation of phenols to carbon dioxide and water, the improvement in the oxygen release ability of the promoter would be a key factor, which also leads to the high catalytic activity. For enhancing the ability of the promoter, the formation of the oxygen vacancies in the lattice might lead to the increase in reducibility and the oxygen mobility. Here, to avoid the metal leaching, the composition of the promoter should be carefully selected. Further investigations would lead to the development of novel catalysts with high efficiency for practical applications.

## Author contributions

NN collected and read papers and wrote the manuscript. AS and P-GC collected papers and contributed to the manuscript writing. NI contributed to the paper design and also wrote the manuscript. All authors read and approved the manuscript.

### Conflict of interest statement

The authors declare that the research was conducted in the absence of any commercial or financial relationships that could be construed as a potential conflict of interest.
